# Characterisation and mechanisms of bradykinin-evoked pain in man using iontophoresis

**DOI:** 10.1016/j.pain.2013.01.003

**Published:** 2013-06

**Authors:** Kathryn J. Paterson, Laura Zambreanu, David L.H. Bennett, Stephen B. McMahon

**Affiliations:** a Wolfson Centre for Age-Related Disease, King’s College London, London, UK; b Nuffield Department of Clinical Neuroscience, University of Oxford, Oxford, UK

**Keywords:** Pain, Bradykinin, Des-Arg9-bradykinin, Iontophoresis, Mast cell

## Abstract

Bradykinin (BK) is an inflammatory mediator that can evoke oedema and vasodilatation, and is a potent algogen signalling via the B1 and B2 G-protein coupled receptors. In naïve skin, BK is effective via constitutively expressed B2 receptors (B2R), while B1 receptors (B1R) are purported to be upregulated by inflammation. The aim of this investigation was to optimise BK delivery to investigate the algesic effects of BK and how these are modulated by inflammation. BK iontophoresis evoked dose- and temperature-dependent pain and neurogenic erythema, as well as thermal and mechanical hyperalgesia (*P *< 0.001 vs saline control). To differentiate the direct effects of BK from indirect effects mediated by histamine released from mast cells (MCs), skin was pretreated with compound 4880 to degranulate the MCs prior to BK challenge. The early phase of BK-evoked pain was reduced in degranulated skin (*P *< 0.001), while thermal and mechanical sensitisation, wheal, and flare were still evident. In contrast to BK, the B1R selective agonist des-Arg9-BK failed to induce pain or sensitise naïve skin. However, following skin inflammation induced by ultraviolet B irradiation, this compound produced a robust pain response. We have optimised a versatile experimental model by which BK and its analogues can be administered to human skin. We have found that there is an early phase of BK-induced pain which partly depends on the release of inflammatory mediators by MCs; however, subsequent hyperalgesia is not dependent on MC degranulation. In naïve skin, B2R signaling predominates, however, cutaneous inflammation results in enhanced B1R responses.

## Summary

An optimised model was developed to investigate in vivo mechanics of bradykinin and analogues in human skin using iontophoresis and an inflammatory model.

## 1 Introduction

Kinin peptides, in particular, bradykinin (BK), are endogenously released from high-molecular-weight plasma globulin kininogen precursors at the site of tissue injury and/or inflammation by kallikrein-mediated hydrolysis [13]. BK was originally identified to cause vascular smooth muscle relaxation and nonvascular smooth muscle contraction (Rocha e Silva in 1962, as referenced [28]). Further investigation in man revealed BK as an algogenic peptide from inflammatory exudates, for example, fluid from arthritic joints and cantharadin blisters [2].

BK acts via 2 G-protein coupled receptors: B1 and B2 receptors with 36% sequence homology [45]. Receptor activation triggers multiple intracellular signalling pathways [13], [56]. B2 receptors (B2R) are constitutively expressed on primary sensory nerve terminals [50], [51]. Meanwhile the presence and functionality of purportedly injury-inducible B1 receptors (B1R) remains controversial.

Acting via the constitutively expressed B2Rs in different tissues, BK has been shown to activate intracellular signalling pathways that result in vasodilatation, neutrophil chemotaxis, and increased vascular permeability [38]. Several studies have investigated the vasodilatory actions of BK in man, mostly in the forearm [9], [29], [48]. BK has been shown to evoke dose-dependent pain [26], [43] and itch [38] when administered via injection, or by iontophoresis [9], [30]. Some of the pain-producing effects of BK probably arise from a direct action on nociceptors (as seen in relatively pure dorsal root ganglion cell cultures), or from an indirect action via other cells expressing BK receptors.

In human skin, mast cells (MCs) are found in the superficial dermal zone adjacent to the dermal-epidermal junction containing granules rich in components such as histamine and heparin. Spatially clustered surrounding capillaries with increased density distally in a glove-and-stocking distribution, there are reputedly approximately 100 MC/mm^2^ in forearm skin [32]. In our experiment we applied BK to an 18-mm-diameter circular area likely to contain more than 25,000 MCs. There is evidence that BK induces MC activation [32]. However, species and tissue may explain discrepancies in the literature, for example, MC activation by BK in murine spleen [32] and rat peritoneum [40], but not in human histaminocytes [40] or epidermal cultures [6], [51]. B2Rs have been reported on human oesophageal MCs [18], but other tissues remain. Given these uncertainties, we wished to evaluate the in vivo contribution of MCs to BK effects in human skin.

B1R expression is reported to be induced on IB4+ nonpeptidergic sensory neurons following tissue injury [13], [19], [51]. A role for B1R in postinjury sensitisation has been demonstrated in rodents using selective antagonists for reversal of thermal and mechanical hyperalgesia in adjuvant and ultraviolet B (UVB)-induced inflammation [50]. However, while B1R agonists have failed to directly stimulate sensory neurons in inflammatory states [15], [46], [55], nonneuronal B1R upregulation suggests an indirect role in nociceptor stimulation [15].The role of the B1R therefore demands further investigation, which we have undertaken here using UVB as an inflammatory stimulus.

In the current experiments, we sought to optimise a method for delivering BK to human skin as well as study mechanisms of BK-evoked pain.

## 2 Methods

Healthy volunteers were recruited to take part in this study. Each participant provided written informed consent prior to commencing each experiment, and the protocol was conducted in accordance with the King’s College London ethics committee (BDM 10/11-84). Individuals with known skin hypersensitivity or dermatological conditions such as eczema or dermatitis were excluded from all experiments. The UVB experiment solely included individuals with skin type 2 or 3 according to the Fitzpatrick scale [3], as established during initial screening. All participants were asked to refrain from analgesic, antiinflammatory, or antihistamine medications for 4 hours, and caffeine or nicotine for 1 hour prior to the experiment.

### 2.1 Iontophoresis

Subjects were comfortably seated in a chair with their arm at the level of the right atrium. Left and right distal volar forearms were alternately tested at sites >4 cm apart. Each experimental site was prepared using an alcohol swab (70% isopropyl alcohol, Uni-Wipe; Universal Hospital Supplies, London, UK). The iontophoresis unit (Phoresor II PM700, Iomed Inc, Salt Lake City, UT, USA) comprised a constant current stimulator and a wire electrode, which runs around the inner circumference of a custom-made perspex ring of 18 mm internal diameter. This perspex chamber was affixed to the skin using a circular adhesive patch around the external rim and filled with 1 mL solution as designated per condition. BK and des-Arg9-BK (both from Sigma, Poole, UK) solutions were freshly prepared as 1 mg/mL solution in 0.9% physiological saline per experiment, and comparison was made to 0.9% saline alone. Inherent to the iontophoresis method, the exact dose of compound cannot be quantified, but is proportional to the charge delivered. That is, iontophoretic delivery of a charged molecule is dependent upon current strength and time of application, following Coulomb’s law; as the product of current and time [52]:

q=I×t,

where q = charge (mC), I = current (mA), and t = time (seconds).

BK, in solution, dissociates into positively charged ions. Therefore, to drive these ions across the skin, the positive lead of the current source was connected to the wire chamber electrode and the negative lead attached to a conductive pad on the outer palm of the ipsilateral hand, which serves as a reference.

### 2.2 Visual analogue scale

Participants were automatically prompted to rate the sensation along a digital visual analogue scale (VAS) at the iontophoretic site every 20 seconds throughout the 8-minute iontophoresis. The VAS was represented by a horizontal bar on a computer screen (0 = no pain, 100 = maximal intensity pain imaginable). The participant clicks the mouse at the point along the line that most accurately describes the sensation at that instant when instructed by the software (Sensation Logger, bespoke, UK). Following each rating, the cursor automatically reverts to 0, and there are no gradations along the line to ensure that the participant rates the sensation as an arbitrary quantification of their maximal perceived pain every time.

### 2.3 Measurement of flare area

The area of solid flare was measured planimetrically through tracing onto acetate. The area was calculated using an 8-spoke wheel (each spoke oriented at 45° intervals) to calculate the total area in square cm, from which the primary area of iontophoresis (18-mm internal diameter circular chamber) was subtracted [49].

### 2.4 Assessment of pain thresholds

Mechanical and thermal pain thresholds were ascertained following selected relevant protocol from the validated and robust battery of standardised Quantitative Sensory Testing as developed by the German Research Network on Neuropathic Pain (DFNS) [53]. These short-form tests were performed by a DFNS-trained experimenter, the same experimenter throughout to avoid interexperimenter variability. Following initial demonstration and instruction for both tests, subjects kept eyes closed throughout testing.

Mechanical pain thresholds were measured at the testing site according to DFNS protocol using a set of 7 custom-made weighted pinprick stimuli (Pinprick, MRC Systems GmbH, Heidelberg, Germany). Each had an identical 2-mm flat contact surface area with rounded edges to avoid physical probe contact that would facilitate nociceptor activation. Each probe exerted fixed force (8, 16, 32, 64, 128, 256, or 512 mN) [4], [42]. The pinpricks were applied individually at a rate of 2 seconds on, 2 seconds off in ascending order until the stimulus was perceived as “sharp,” then applied in descending order until perceived as “blunt.” The threshold was defined by the “method of limits” as the geometric mean of 5 measurements of sharp and blunt in a sequential series of ascending and descending stimuli.

Heat pain threshold was derived as the arithmetic mean of 3 consecutive measurements using a thermal sensory analyser (TSA 2001-II; Medoc Ltd, Ramat Yishai, Israel) thermode held against the skin [1], [25]. Thresholds were obtained using ramped stimuli (1 °C/s), which terminated when the subject pressed a button to indicate first percept of pain. Temperature of the thermode declined to baseline temperature of 32 °C (centre of neutral range) at a rate of ∼5 °C/s and remained at 32 °C during 10-second interstimulus intervals. The contact area of the thermode was 256 mm^2^ (16 × 16 mm). A maximal cut-off temperature of 52 °C was employed whereby the thermal sensory analyser would automatically return to baseline temperature. All subjects were unaware of the timing of initiation of temperature increase and the interstimulus intervals. Thermal testing was first demonstrated over an area that was not used for testing during the experiment, that is, the dorsum of the hand. The skin was initially inspected for lesions or scarring, which may alter sensory perception.

**Fig. 1:**
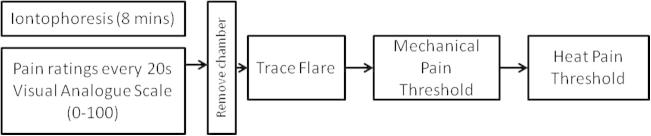
Basic process common to all experiments described.

During all experiments, participants were blinded to the current and nature of the solution being administered (ie, whether vehicle control or test solution). The experimenter was also blinded as to the solution for each condition. All conditions were applied in a pseudorandomised sequence. Following each iontophoresis session of a test solution, the chamber was removed, flare measured, and sensory tests conducted as per Fig. 1.

### 2.5 Assessment of the effects of iontophoretic current on BK-evoked pain

Forearm skin was maintained at 35 °C using an infrared radiant lamp and monitored throughout the experiment using a noncontact convergent infrared laser thermometer (DT-8861; ATP Instrumentation Ltd, Ashby-de-la-Zouch, UK) whilst temporarily averting the lamp. Iontophoresis of test solutions using currents of 0.4, 0.8, and 1.2 mA over 8 minutes was performed. BK or saline were iontophoresed at all doses to independent sites in random sequence. Following iontophoresis, the chamber was removed, the flare measured, and sensory tests conducted as per Fig. 1.

### 2.6 Assessment of the effects of skin temperature on BK-evoked pain

Forearm skin temperature (25°, 30°, 35°, 40 °C) was modulated using either a gel-filled cooling refrigerant pack at an appropriate temperature or an infrared lamp, as appropriate. Temperature was monitored throughout the 8-minute iontophoretic protocol using a convergent infrared laser beam thermometer (DT-8861, ATP Instrumentation Ltd) whilst temporarily removing either the heating or cooling source. At target temperature, iontophoresis was initiated at 1.2 mA over 8 minutes. In randomised order, BK (1 mg/mL in saline) and saline were iontophoresed at all test temperatures to independent volar forearm sites.

### 2.7 Mast cell degranulation

Compound 4880 (5 mM in deionised water; Milli-Q; Millipore, Livingston, UK), known to degranulate MCs, was iontophoresed at 0.4 mA × 8 minutes using an 18-mm-diameter perspex chamber to the volar forearm bilaterally at the same position equidistant between antecubital fossa and wrist. Iontophoresis was performed 5 times in total, at time 0, and then at 3 hours, 6 hours, 9 hours, and 24 hours to ensure MC depletion [20]. As a measure of depletion, area of flare was measured immediately following each session once the chamber and solution had been cleared from the site and the exact location of the chamber marked onto the skin.

One hour following the final flare measurement (at 24 hours from the beginning of the experiment), the area was left for 3 hours prior to iontophoretic application to the same site of either BK (1 mg/mL in 0.9% saline) or saline using 1.2 mA × 8 minutes, maintained at 35 °C using infrared heat lamp as described above.

### 2.8 Comparison of BK and des-Arg9-BK in naïve skin

B2R selective agonist BK, B1R selective agonist des-Arg9-BK (both 1 mg/mL in saline) and saline solutions were iontophoresed in random sequence to independent forearm sites sequentially alternating arms. All iontophoresis sessions were conducted maintaining skin temperature at 35 °C using a current of 1.2 mA × 8 minutes.

### 2.9 Comparison of BK and des-Arg9-BK in UVB-irradiated skin

UVB irradiation was administered using Philips (Guilford, Surrey, UK) TL01 fluorescent bulbs with a maximum wavelength of 311 nm. The irradiance output was measured with a photometer (IL1400A with SEL240/UVB-1/TD filter; ABLE Instruments and Controls, Aberdeen, UK) placed at the distance of the exposed skin. This reading was used to calculate the time needed to deliver an appropriate UVB dose. In this manner, a skin-type-specific UVB dose irradiation series was given by exposing volar forearm skin to 6 areas of 16 × 16 mm areas of increasing UVB exposure given by the equation: Energy = Lamp Irradiation × Time [60], as described elsewhere [7]. At 48 hours post irradiation, each square was assessed for mean erythematic dose (MED). This same dose of UVB irradiation was then administered to 3 naïve volar forearm sites bilaterally. At 48 hours post irradiation, the B1R selectivity experiment was repeated at the 3 irradiated sites.

### 2.10 Data evaluation

All statistical analyses were performed using GraphPad Prism (La Jolla, CA, USA).

All pain rating data were presented both as mean rating per 20-second timepoint and mean of individual area under the curve (AUC) data for each condition. All data were analysed for their distribution properties using the Shapiro-Wilk test for normality.

Mechanical pain threshold data were found to be normally distributed in log-space and therefore transformed logarithmically before statistical analysis [53]. Differences between normally distributed vehicle and test compound data for each condition were compared using a repeated-measure 2-way analysis of variance (ANOVA) with Bonferroni post hoc analysis. Log-data of thresholds were re-transformed to linear values representing the original unit of the test and presented as such. All data are presented as mean ± SEM unless otherwise stated.

## 3 Results

### 3.1 Iontophoretic delivery of BK to skin evokes ongoing pain as well as thermal and mechanical hyperalgesia

As BK is a positively charged molecule, we wanted to establish a protocol for the iontophoretic delivery, which would enable controlled application of the molecule to the skin, in order to study evoked pain and the sensitising effects of BK. Increasing doses of iontophoretic current (from 0.4 to 1.2 mA) were used to enhance BK delivery. BK iontophoresis rapidly produced pain within the first minute of iontophoresis. Interestingly, there were 2 peaks of BK-evoked pain, at approximately 2 minutes and 5 minutes from the start of iontophoresis, and following the second peak there was a decline in the BK response.

**Fig. 2:**
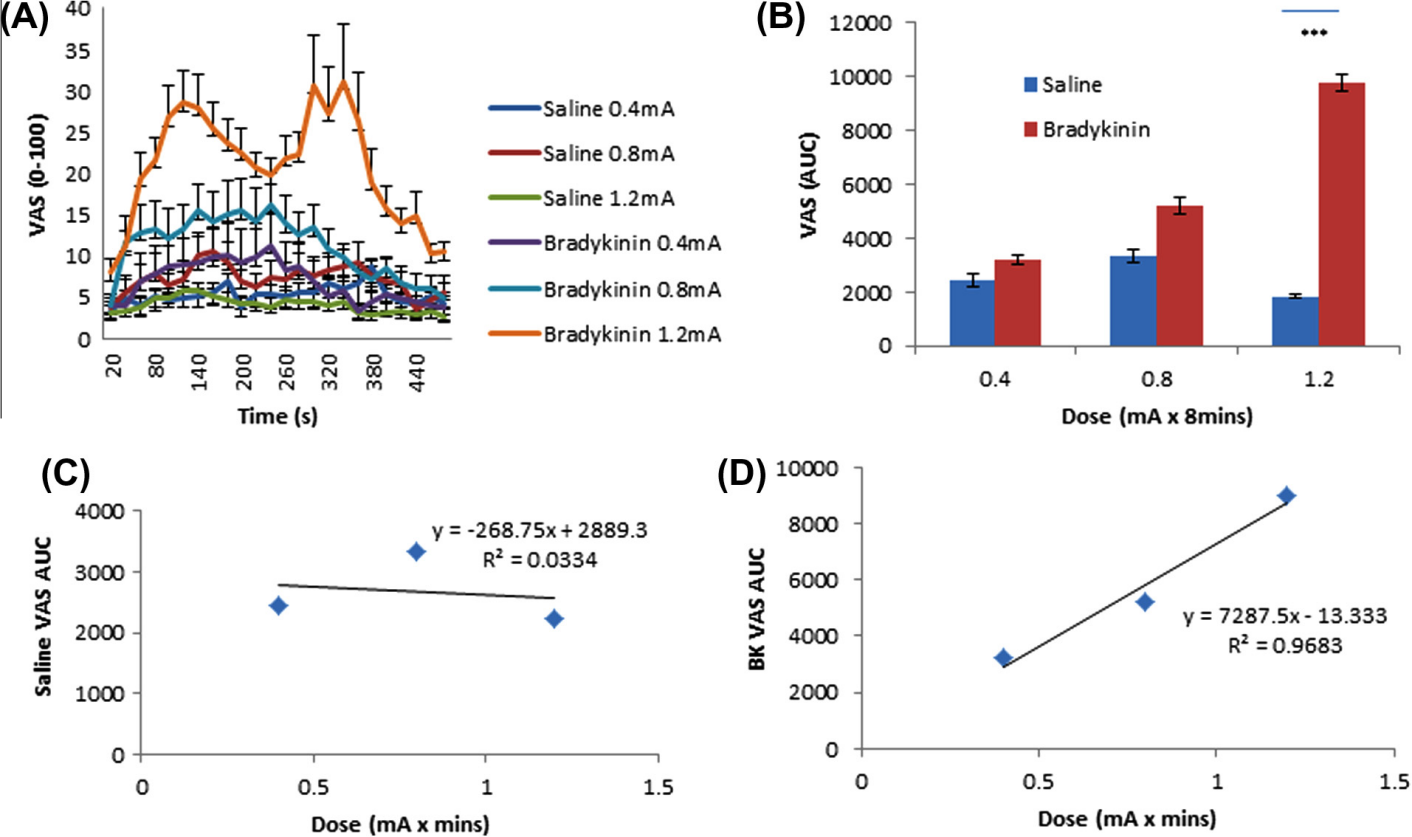
Investigating dose-dependent pain following bradykinin (BK) administration. (A) Pain ratings during iontophoresis of BK and saline vehicle. Displayed as average at each 20-second timepoint. (B) Area under the curve (AUC). Pain ratings during 8-minute iontophoresis compound administration. Results displayed as average n = 10 with SEM. ^∗∗∗^*P *< 0.001, 2-way repeated-measures analysis of variance with Bonferroni post hoc analysis; n = 10. (C) Saline visual analogue scale (VAS) AUC data plotted at each dose to demonstrate any linearity with dose response; *R*^2^ = 0.0334. (D) BK VAS AUC data plotted at each dose to demonstrate any linearity with dose response; *R*^2^ = 0.9683.

There was a clear current dose-dependent effect on pain as rated on the VAS scale during the 8-minute period of iontophoresis (Fig. 2A). Using AUC analysis, the effect of BK was significantly different from saline using a current of 1.2 mA (Fig. 2B; *P *< 0.001, n = 10, 2-way ANOVA with Bonferroni post hoc analysis). This dose-dependent effect was confirmed by the demonstration of linear significance of pain responses to BK (Fig. 2D) not seen with saline (Fig. 2C).

**Fig. 3:**
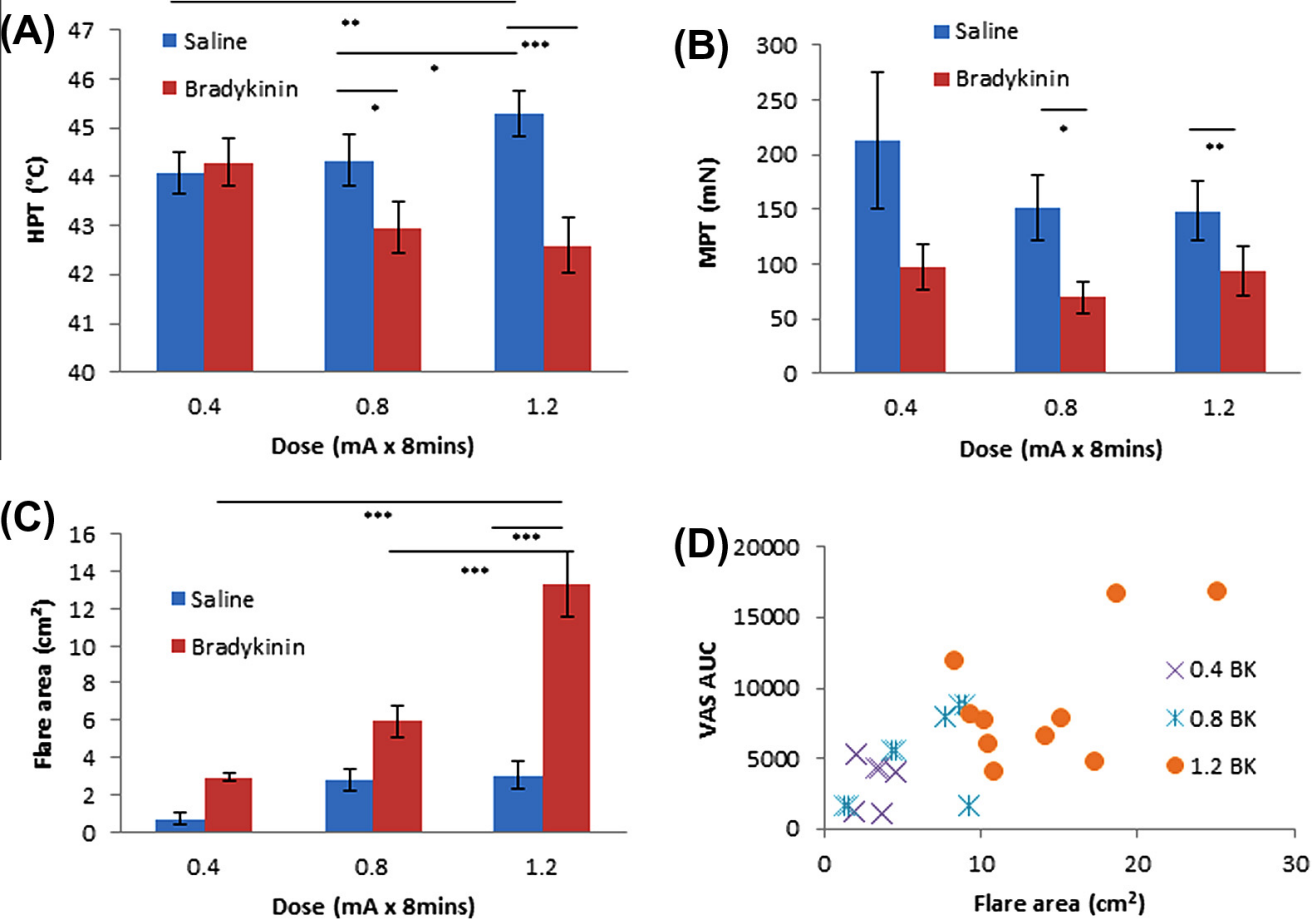
Quantitative testing following 8-minute application. (A) Heat pain thresholds (HPT). (B) Mechanical pain thresholds (MPT) were logarithmically transformed and paired *t*-tests performed. 0.8 bradykinin (BK) vs saline *P *= 0.044, 1.2 mA *P *= 0.01. (C) Area of flare following each treatment. n = 10 with SEM. (A, C) Two-way repeated-measures analysis of variance with Bonferroni post hoc analysis. VAS, visual analogue scale; AUC, area under the curve.

Thermal pain thresholds were reduced after BK treatment (Fig. 3A). The effect was dose dependent and was seen following doses of 0.8 mA and 1.2 mA. Interestingly, the highest dose of saline produced a significant increase in heat pain threshold.

Following BK administration, a trend of reduced mechanical pain threshold was demonstrated in comparison to saline control under the same conditions (Fig. 3B). This mechanical hypersensitivity was greatest with 1.2-mA currents (*P *= 0.01, n = 10, paired *t*-test). The area of flare generated by application of BK was dose-dependently greater than that produced by vehicle control under all conditions (Fig. 3C) (*P *< 0.001, n = 10, 2-way ANOVA with Bonferroni post hoc analysis). This effect is highly correlated at the higher doses, as demonstrated by Fig. 3D (0.4 mA *R*^2^ = 0.0061; 0.8 mA *R*^2^ = 0.1821; 1.2 mA *R*^2^ = 0.3394).

### 3.2 Skin temperature influences thermal and mechanical sensitisation following BK delivery

**Fig. 4:**
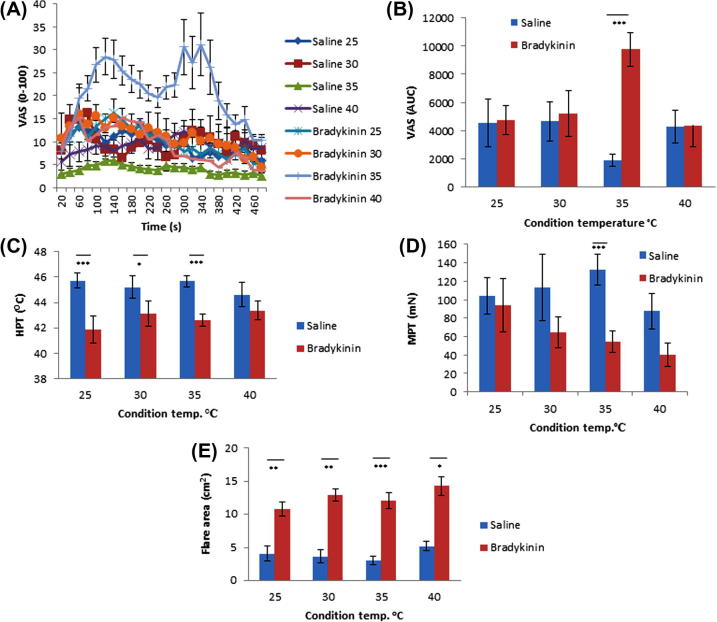
Iontophoresis maintaining constant temperature. (A) Pain ratings during bradykinin application. (B) Pain ratings during saline application. (C) Area under the curve (AUC) of average visual analogue scale (VAS) at each 20-second timepoint for each condition. Results displayed as average n = 10 with SEM. One-way analysis of variance (ANOVA) with Bonferroni post hoc analysis, ^∗∗∗^*P *< 0.001. (D) Heat pain thresholds. (E) Mechanical pain threshold. (F) Flare area. (A, C) Two-way ANOVA with Bonferroni post hoc analysis. ^∗^*P *< 0.05; ^∗∗^*P *< 0.01; ^∗∗∗^*P *< 0.001. (E) Paired t-tests performed on log-transformed data. HPT, heat pain threshold; MPT, mechanical pain threshold.

To determine impact of skin temperature on the effects seen by the exogenous application of BK, the site was maintained at a constant temperature (25°, 30°, 35°, 40 °C) throughout the 8-minute iontophoretic delivery. During the administration of both solutions, dose remained constant at 1.2 mA. Pain ratings during iontophoresis were virtually indistinguishable between BK and saline control, whilst skin temperature was maintained at 25°, 30°, and 40 °C (Fig. 4A and B). Only while skin temperature was held at 35 °C was a biphasic response demonstrated during BK delivery (Fig. 4A). Using AUC (Fig. 4B), the pain ratings during BK administration were shown to be significantly increased at 35 °C compared to saline vehicle control (*P *< 0.001, n = 10, 2-way ANOVA with Bonferroni post hoc analysis), which is diminished at this temperature.

The sensitising and flare-inducing effects of BK, however, were not so temperature dependent. We observed a clear and statistically significant thermal sensitisation at all skin temperatures except at 40 °C (Fig. 4C). Mechanical sensitisation was significant at 35 °C and 40 °C and approaches significance at 30 °C (Fig. 4D). While BK consistently produced greater flare than saline under each condition, this was again maximal at the physiological temperature of 35 °C (Fig. 4E; *P *< 0.001, n = 10, 2-way ANOVA with Bonferroni post hoc analysis).

Together, these results show that BK iontophoresis results in thermal and mechanical hyperalgesia and a clear flare response, and at higher doses, pain. Effects were optimal at a skin temperature of 35 °C, close to that typically found under normal physiological circumstances, and using an iontophoretic current of 1.2 mA. These optimised conditions were therefore used to further investigate the action of BK in vivo.

### 3.3 Effects of MC degranulation on evoked pain, flare, and sensitivity

**Fig. 5:**
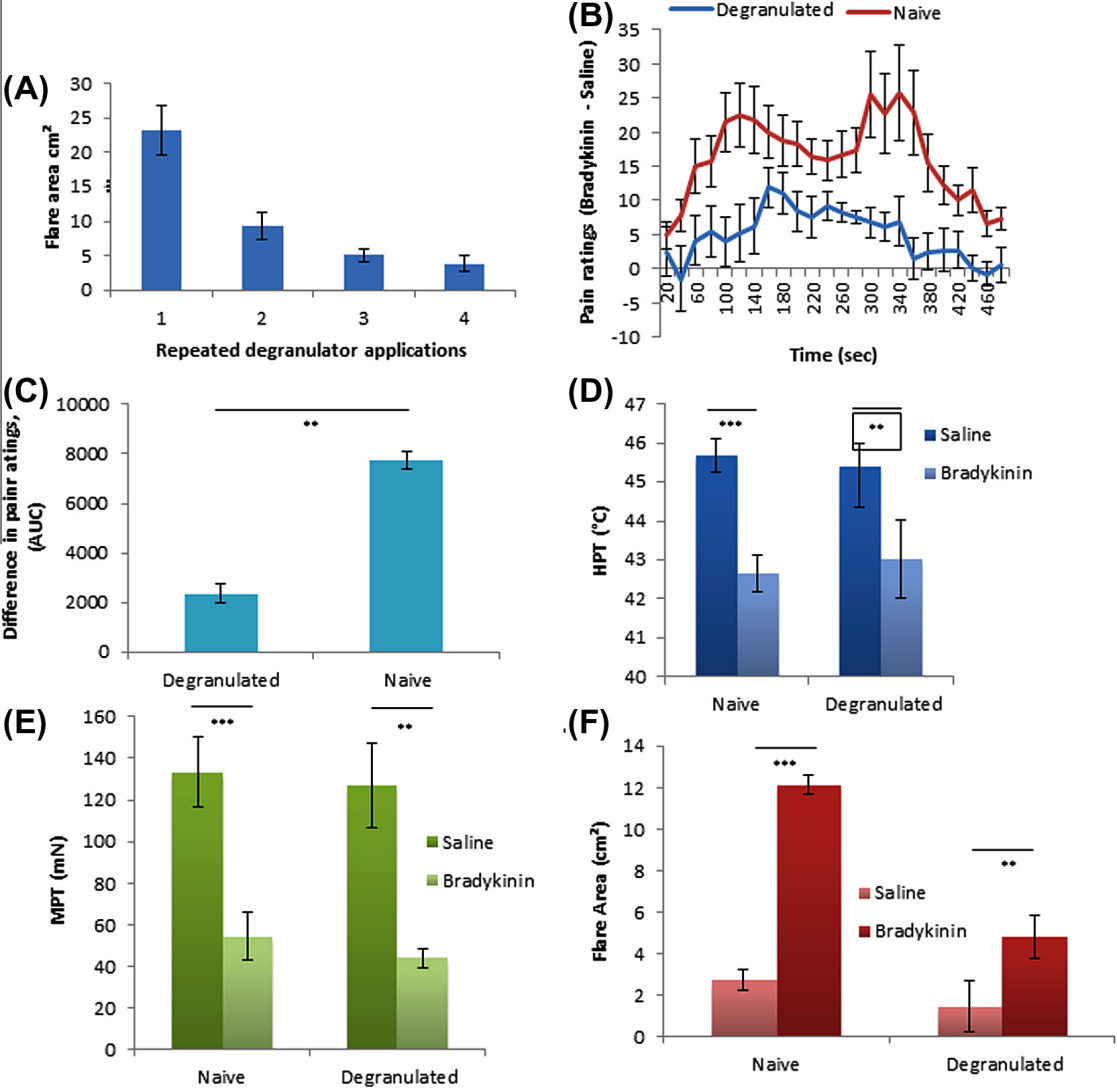
Mast cell degranulation. (A) Mast cell degranulation by repeated iontophoretic application of compound 4880. Extent of mast cell degranulation indicated by size of flare outside of primary site. (B) Difference between pain ratings during iontophoresis of bradykinin (BK) and saline in both naïve and mast cell degranulated skin. (C) Area under the curve (AUC) for the difference between pain ratings as shown in (B); paired *t*-test; *P *= 0.02. Quantitative sensory testing following iontophoretic application of saline or BK in both naïve and mast cell degranulated skin. (D) Heat pain thresholds (HPT) naïve *P *< 0.001, degranulated *P *= 0.039. (E) Mechanical pain thresholds (MPT) naïve *P *< 0.001, degranulated *P *= 0.002. (F) Area of flare naïve *P *< 0.001, degranulated *P *= 0.035. A and C both use one-way analysis of variance with Bonferroni post hoc analysis.

Gradual MC degranulation over time was confirmed by measurement of flare following each application of compound 4880 (Fig. 5A). Wheal responses were initially uniform and large, but with subsequent applications of compound 4880 they were fragmented and greatly reduced. Pain ratings were recorded during both BK and saline iontophoresis under optimised conditions (1.2 mA × 8 minutes at 35 °C) in both naïve and MC degranulated skin. For clarity, the difference between BK and saline pain ratings on a VAS scale (BK minus saline at each 20-second timepoint) are compared in both naïve and degranulated skin (Fig. 5B). In naïve skin, a characteristic biphasic response was seen, with peaks at approximately 120 and 300 seconds. However, in degranulated skin, considerably less pain was reported throughout the trial, and the later peak seen in control was absent. The difference (BK − saline) in naïve and degranulated skin was found to be significant by AUC analysis (Fig. 5C, *P *= 0.02, n = 10, paired *t*-test).

The thermal hyperalgesia elicited by BK, however, was indistinguishable in naïve and degranulated skin (Fig. 5D, *P *= 0.039 in MC degranulated skin [BK vs control], *P *< 0.001 in naïve, one-way ANOVA).

Similarly, mechanical hyperalgesia was seen following BK iontophoresis to a similar degree in both naïve and degranulated skin (Fig. 5E). Therefore, the sensitising effects of BK, unlike the pain-producing effects, are unaffected by depletion of MC products.

BK-evoked flare exceeded that caused by saline in both naïve and degranulated skin (Fig. 5F). However, in the absence of MC products, flares were significantly smaller (*P *< 0.001 in naïve, *P *= 0.035 in degranulated skin, n = 10, one-way ANOVA with Bonferroni post hoc analysis).

### 3.4 The effects of the B1 receptor agonist des-Arg9-BK in naïve skin

BK, known to have great affinity for B2R, was compared under optimised conditions to the B1R agonist des-Arg9-BK in naïve skin.

**Fig. 6:**
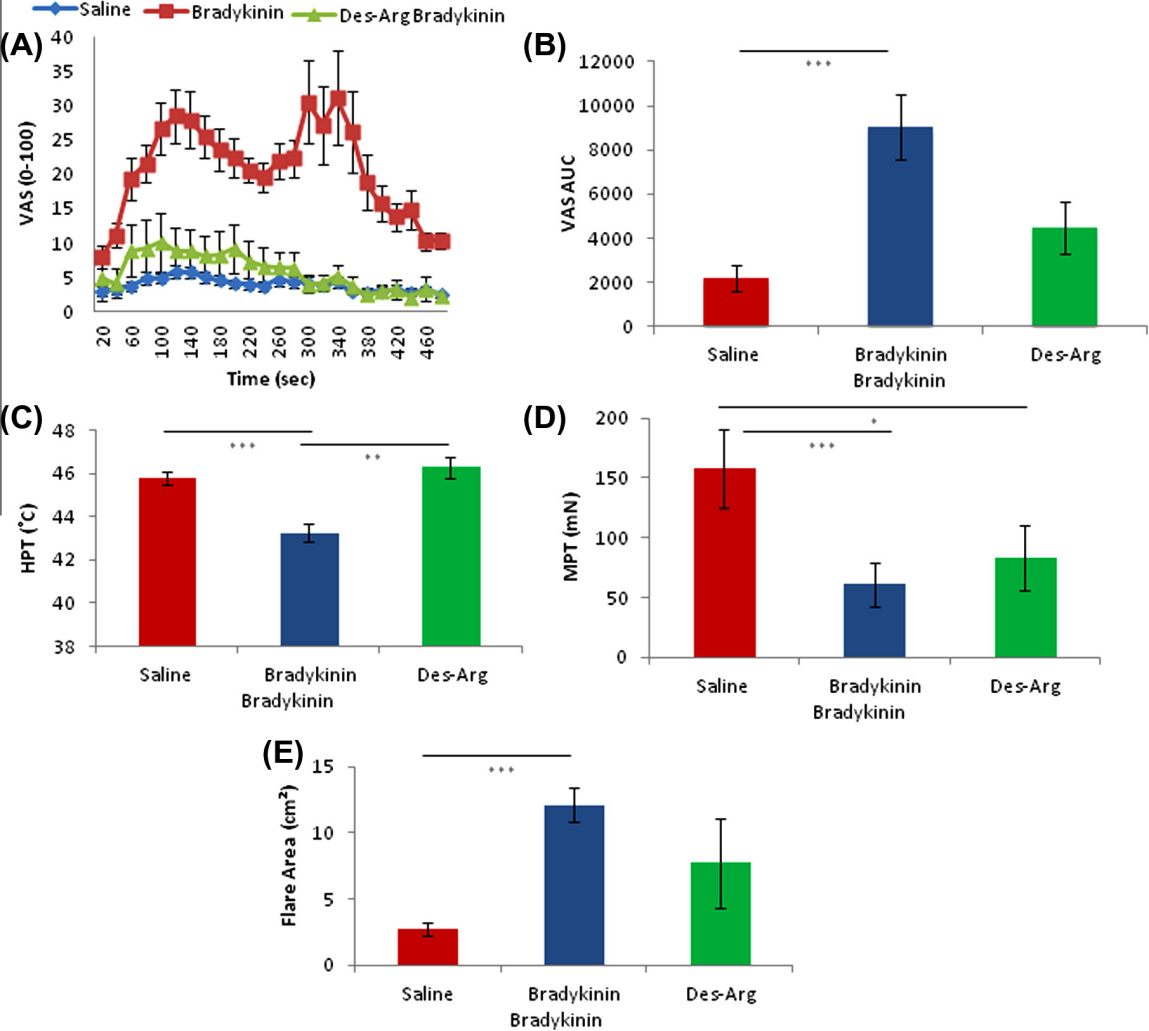
Application of a B1R agonist in naïve skin. (A) Pain ratings during 8-minute iontophoresis of saline, bradykinin (BK), and des-Arg9-BK using a visual analogue scale (VAS; 0-100). (B) Area under the curve (AUC) from Fig 8A. Saline vs BK *P *< 0.001, BK vs des-Arg9-BK *P *= 0.02, n = 10, one-way repeated-measures analysis of variance (ANOVA) and Bonferroni post hoc analysis. Comparing BK, Des-Arg BK, and saline vehicle control in naïve skin. (C) Heat pain threshold (HPT). Saline vs BK *P *< 0.001, BK vs Des-Arg9-BK *P *= 0.018. (D) Mechanical pain threshold (MPT) saline vs BK *P *< 0.001, saline vs Des-Arg9-BK *P *= 0.04. Data log transformed and paired *t*-tests performed. (E) Flare area saline vs BK *P *< 0.001. (C) and (D) both use one-way ANOVA with Bonferroni post hoc analysis.

The characteristic twin peak time profile of pain rated during iontophoresis of BK was not seen during application of the B1 receptor agonist des-Arg9-BK (Fig. 6A). Quantified by AUC (Fig. 6B), BK evoked significantly greater pain ratings over time than both saline (*P *< 0.001) and des-Arg9-BK (*P *= 0.02; n = 10, one-way ANOVA with Bonferroni post hoc analysis). In naive skin, des-Arg9-BK evoked painful sensation not statistically different from saline.

Des-Arg9-BK iontophoresis did not induce the thermal sensitisation seen with BK (Fig. 6C).

Mechanical hyperalgesia was observed by both BK and des-Arg9-BK iontophoresis using pinprick stimuli (Fig. 6D).

Area of flare evoked by des-Arg9-BK was highly variable amongst the group of 10 subjects tested. The result was that the flare elicited by the B1 agonist in naïve skin was statistically unchanged from flare evoked by saline, while BK, as expected, evoked a clear and significant flare (Fig. 6E).

### 3.5 The effects of the B1 agonist des-Arg9-BK in skin inflamed with UVB

We compared the effects of B1 and B2 ligands (des-Arg9-BK and BK, respectively) in UVB-irradiated skin using methods previously described [7], [16], based upon previous reports that the B1 receptor is induced under such conditions. We compared iontophoretic application of des-Arg9-BK with BK in a group of subjects who had received a modest dose (1 Minimal Erythema Dose (MED)) of UVB irradiation. These were the same subjects who were tested with des-Arg9-BK in normal skin.

**Fig. 7:**
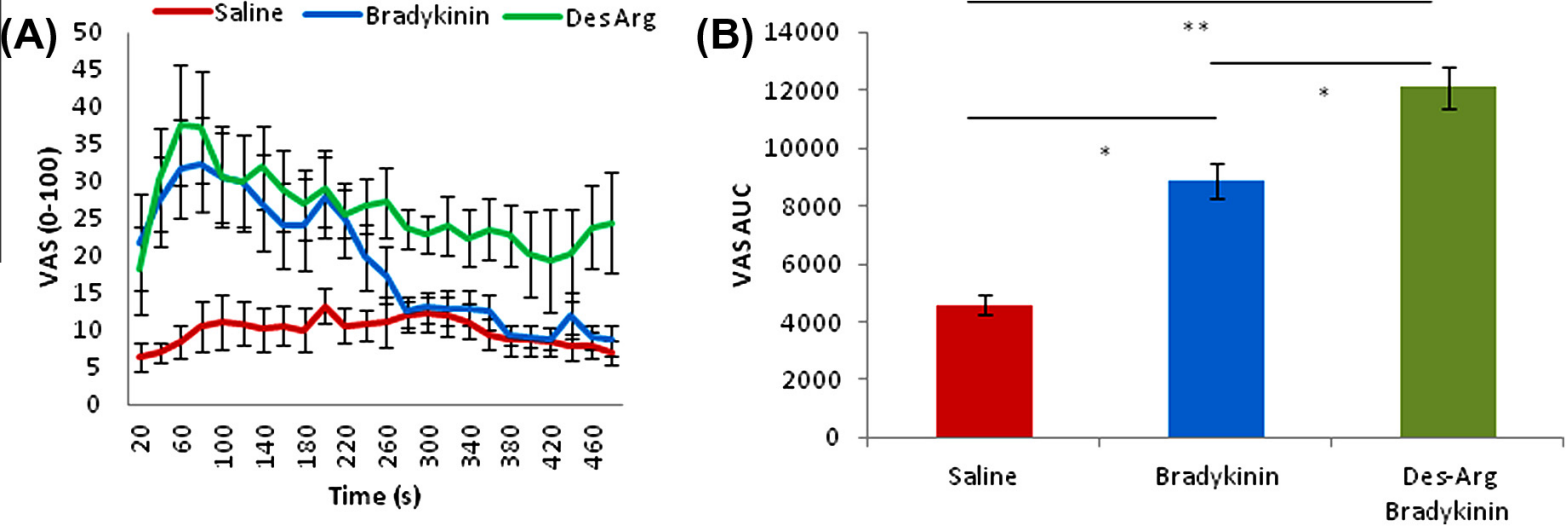
Investigating B1R selective agonist in ultraviolet-irradiated skin. (A) Pain ratings during iontophoresis, using visual analogue scale (VAS) at 20-second intervals. (B) Area under the curve (AUC) of pain ratings. Saline vs bradykinin (BK) (*P *= 0.04), Saline vs des-Arg9-BK (*P *= 0.004), BK vs des-Arg9-BK (*P *= 0.014), one-way analysis of variance + Bonferroni post hoc analysis n = 10.

Pain ratings during iontophoresis were, on average, higher with des-Arg9-BK compared with BK in inflamed skin, and, of course, in contrast to the effects seen in naive skin (Fig. 7). Total VAS scores were similar in naïve and inflamed skin, but the response in inflamed skin appeared monophasic rather than biphasic. Des-Arg9-BK evoked about the same peak level of pain, but this was more sustained throughout the application period.

**Fig. 8:**
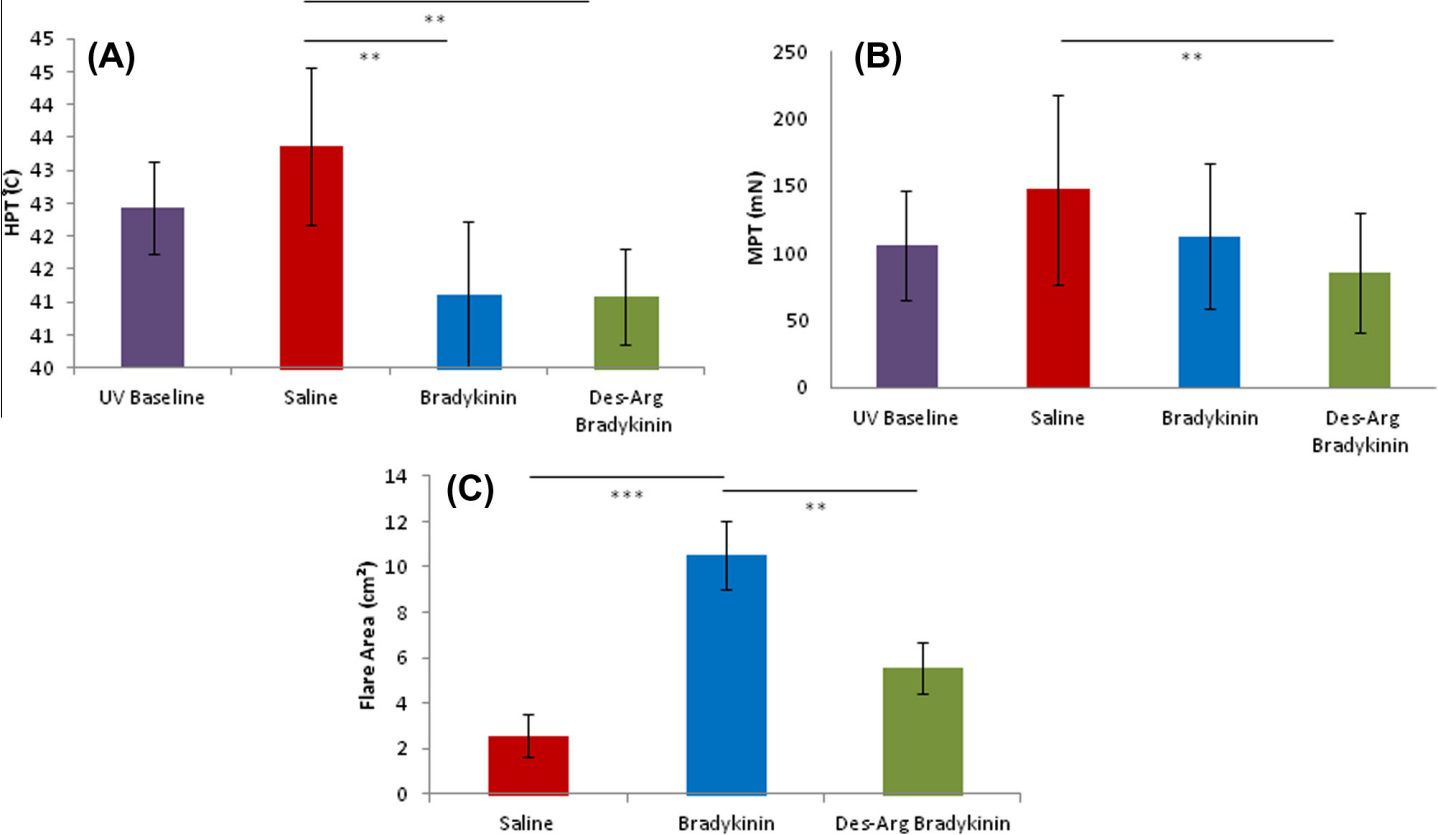
Comparing saline, BK, and des-Arg9-BK in inflamed skin. (A) Heat pain threshold (HPT). Saline vs des-Arg9-BK *P *= 0.024, saline vs BK *P *= 0.024. (B) Mechanical pain threshold (MPT). Saline vs Des-arg *P *= 0.003. (C) Area of flare. Saline vs BK *P *< 0.001, BK vs des-Arg9-BK *P *= 0.026. (A) and (C) analysed using one-way repeated-measures analysis of variance + Bonferroni post hoc analysis. (B) Analysed by paired *t*-tests on log-transformed data.

Also, in contrast to normal skin, BK and des-Arg9-BK produced similar reductions in heat pain thresholds in inflamed skin. This was not seen with saline iontophoresis (Fig. 8A; *P *= 0.024, n = 10, one-way ANOVA with Bonferroni post hoc analysis). The magnitude of kinin-induced hyperalgesia is lower than that seen in naïve skin, which may be explained by the heat pain thresholds already being reduced in UVB inflamed skin.

Des-Arg9-BK evoked a modest but statistically significant mechanical hyperalgesia in inflamed skin (Fig. 8B; *P *= 0.003, n = 10, one-way ANOVA with Bonferroni post hoc analysis), again in contrast to its effects in normal skin. BK in inflamed skin elicited only a small average mechanical hyperalgesia and this was not significant (Fig. 8B), perhaps again because mechanical pain thresholds are already lower in inflamed skin and there is therefore a smaller “window” in which to observe kinin effects. BK and des-Arg9-BK both produced significant flare responses in inflamed skin (Fig. 8C; *P *< 0.001, n = 10, one-way ANOVA with Bonferroni post hoc analysis). BK-induced flares were comparable to those seen in normal skin (in terms of area). Flare evoked by des-Arg9-BK was less than that produced by BK, but was significantly greater than that seen with saline (*P *= 0.026, n = 10, one-way ANOVA with Bonferroni post hoc analysis).

In summary, these results reveal a clear emergence of B1-mediated algesic effects in inflamed skin that is not observed in normal skin.

## 4 Discussion

We have developed an experimental human pain model employing BK iontophoresis in order to dissect the mechanisms by which this molecule evokes pain and sensitisation. BK evoked pain in a dose- and temperature-related manner during iontophoresis. This was associated with the development of a cutaneous flare and thermal hyperalgesia as seen previously [43], in addition to mechanical hyperalgesia also seen here. The flare and BK-evoked pain were partly dependent on MCs. The induced mechanical and thermal hyperalgesia, however, were MC independent. In naive skin, des-Arg9-BK, which shows selectivity for the B1R (vs BK, which has selectivity for the B2R) evoked minimal pain and had only mild sensitising effects. The effects of des-Arg9-BK were, however, potentiated following UVB irradiation of the skin. This is, therefore, a robust model system in which to probe the sensitising effects of BK, and emphasises the enhanced role of B1Rs in inflammatory pain states.

Being a charged molecule, BK lends itself to administration by the noninvasive method of iontophoresis. The precise dose delivered during iontophoresis, while unknown, is proportional to the magnitude and duration of applied current. We chose this method of application because achieving a steady state level of BK in the skin by continual application is arguably more representative of the physiological inflammatory condition than bolus injection, which likely diffuses rapidly from the area.

We have used a range of doses and temperature conditions to demonstrate a dose-dependent induction of pain and sensitisation by BK; these effects were maximal at the highest current used (1.2 mA) and at 35 °C. However, one important finding is that the pain-producing effects of BK require higher doses than the sensitising effects; in particular, the thermal hyperalgesic effect. A dose-dependent algogenic effect of BK is supported by previous studies following injection or microdialysis in human skin [22], [36], [43]. The effect of temperature on BK-evoked pain and sensitisation was complex. BK robustly evoked pain at 35 °C, but not at lower temperatures. In light of evidence that prolonged (30 minutes) exposure to the full range of temperatures used here were not inherently painful [10], we can be confident that any effect seen is attributable to BK. Evidence suggests that nociceptor excitation by BK is mediated via transient receptor potential V1 (TRPV1) and TRPA1 ion channels [5]. BK has a striking ability to induce thermal sensitisation of nociceptive afferents [41]. BK has also been demonstrated to lower the thermal activation threshold of TRPV1 to below 37 °C [58]; these interactions may explain the temperature dependence of BK-evoked pain. Interestingly, at a skin temperature of 40 °C, BK at the same dose did not evoke pain. This may feasibly be due to temperature-enhanced vasodilatation increasing compound clearance or enhanced enzymatic cleavage. The temperature dependence of the sensitising effects of BK were quite distinct: whilst thermal and mechanical hyperalgesia are both clearly observed at 35 °C, thermal hyperalgesia is also seen at the lower temperatures tested (25° and 30 °C), but not at the higher temperature of 40 °C. Meanwhile, mechanical hyperalgesia shows the opposite pattern (sensitisation observed at temperatures above but not below 35 °C). The molecular explanation for these differences is not clear, but it is known that the mechanical and thermal transducers are different and subject to independent controls.

The time course of pain evoked by continuous iontophoresis of BK was interesting. We observed 2 peaks in pain ratings, at approximately 120 seconds and 300 seconds following onset of iontophoresis. Each peak was followed by a decline in ratings, which may be explained by tachyphylaxis, a phenomenon consistently reported by previous studies. Declining pain reports are unlikely to be explained by psychological acclimatisation, BK metabolism, or dilution of the peptide in accumulating fluid. Desensitisation of the B2R receptor, however, is another possibility, as demonstrated in vitro [24]. The second peak in evoked pain is likely to represent the engagement of a different mechanism, as discussed below.

There is debate as to what extent BK evokes mechanical hyperalgesia. Following intradermal injection, no evidence of mechanical hypersensitivity was found in human volunteers [43], although mechanical hypersensitivity has been reported following intradermal injection in rat [35]. We found a clear mechanical hyperalgesia following iontophoresis of BK. This robust effect compared to the conflicting results following injection may be as a consequence of the more prolonged BK application that is possible with iontophoresis. This mechanical hyperalgesia may be as a consequence of peripheral or central sensitization. Certain primary afferent populations (such as feline articular afferents) have been shown to be sensitised to mechanical stimuli in response to BK [47]; sensitisation of cutaneous nociceptors to mechanical stimuli following BK has not, however, been observed either in rat nociceptors [61], nonhuman primates [36], or in recordings from human Mechano-heat sensitive C (CMH) fibres [54]. The mechanical hyperalgesia evoked by BK may, rather than a direct nociceptor effect, be mediated by the BK-evoked release of other mediators such as Adenosine Tri-Phosphate (ATP) [17] or prostanoids [19], [40].

Following iontophoresis of BK, we observed thermal hyperalgesia, as others have previously reported following injection of this agent [43]. There is strong evidence for a peripheral component of such hyperalgesia. That is, BK has been shown to sensitise rodent [37], nonhuman primate [36], and human [54] nociceptors to thermal stimuli. BK has been shown to enhance heat-induced currents in cultured sensory neurons, an effect dependent on activation on PKC epsilon [11]. The lowering of the threshold of TRPV1 to thermal stimuli by BK has been discussed above and may be partly mediated by the release of lipoxygenase products of arachidonic acid in response to BK [57].

Some of the effects of BK on nociceptive afferents may be indirect and mediated via other cells, such as MCs [21], [31]. MCs are a well-recognised source of mediators such as histamine, the release of which can be triggered under a variety of circumstances, including inflammation. In our experiments, MC degranulation significantly reduced the early phase of pain evoked by BK iontophoresis, but did not prevent the sensitising effects of BK. There is a mixed literature on the effects of BK on MCs. BK is well recognised to evoke a flare and wheal response in human skin [34], [59] and indeed, was seen in the present study. The flare response has been reported to be abolished by both cyclooxygenase [14] and histamine receptor inhibitors [23] and by compound 4880 treatment [59]. However, BK is also reported not to directly stimulate release of histamine from human skin MCs in vitro [6], [12], [39], and has been found to increase permeability in vivo via a largely histamine-independent mechanism [26], [33]. One explanation for these different findings is that MCs behave differently in vivo and in vitro. Alternatively, BK may activate MCs indirectly in vivo, via yet another cell type.

We did not see any effect of MC degranulation on the sensitising action of BK, further reinforcing the notion that separate mechanisms may be responsible for the sensitising and pain-producing effects. MC products can contribute to nociceptor sensitisation under certain circumstances [21], but there is no direct evidence that this is the case when BK is the sensitising agent. The most parsimonious explanation is that BK acts to directly sensitise nociceptors at low doses, and at higher doses, releases mediators from other cell types, including MCs that activate nociceptors and cause pain. Other studies have dissociated pain-producing and flare responses to BK [33], [34], as we too found here.

The role of B1 receptors is controversial. Under normal conditions, most reports claim that nociceptors express B2 but not B1 receptors (but see [62]). B1 receptors are, however, upregulated in nociceptors under inflammatory conditions [27]. Des-Arg9-BK is a known B1 receptor selective agonist [8], [44] with 10,000× lower affinity than that of BK for the constitutively expressed B2 receptors. Our studies using these 2 agonists support the conventional rule. That is, that in normal skin, the B1 agonists produced much less pain and thermal sensitisation than seen with the B2 agonist. In contrast, we found that thermal and mechanical hyperalgesia were induced to a similar degree by both agonists in UVB-inflamed skin. These latter observations also suggest that effects of des-Arg9-BK in normal skin were unlikely to be due to other factors such as inadequacy of delivery.

Our data also inform on the role of B1 and B2 receptors in vascular responses to BK. We found that des-Arg9-BK produced a variable flare response in normal skin and a more consistent effect in inflamed skin. One other study has compared these agonists in human skin using a microdialysis technique, and also compared normal and UVB-inflamed skin [22]. That study also concluded that neuronal rather than vascular induction of B1Rs was more important in inflamed states.

In conclusion, this study reports a reliable and noninvasive method for assessing the algogenic effects of BK in humans. We have been able to dissociate several distinct effects of BK, including the effects of B1 vs B2R agonists. This may be particularly useful for analgesic drug development programmes because there is an ongoing interest in developing B1 antagonists as analgesic drugs.

## Conflict of interest statement

The authors have no conflicts of interest to report.

## References

[1] Arendt-Nielsen L., Yarnitsky D. (2009). Experimental and clinical applications of quantitative sensory testing applied to skin, muscles and viscera. J Pain.

[2] Armstrong D., Jepson J.B., Keele C.A., Stewart J.W. (1957). Pain-producing substance in human inflammatory exudates and plasma. J Physiol.

[3] Astner S., Anderson R.R. (2004). Skin phototypes 2003. J Investig Dermatol.

[4] Baumgärtner U., Magerl W., Klein T., Hopf H.C., Treede R.-D. (2002). Neurogenic hyperalgesia versus painful hypoalgesia: two distinct mechanisms of neuropathic pain. PAIN®.

[5] Bautista D.M., Jordt S.-E., Nikai T., Tsuruda P.R., Read A.J., Poblete J., Yamoah E.N., Basbaum A.I., Julius D. (2006). TRPA1 mediates the inflammatory actions of environmental irritants and proalgesic agents. Cell.

[6] Benyon R., Lowman M., Church M. (1987). Human skin mast cells: their dispersion, purification, and secretory characterization. J Immunol.

[7] Bishop T., Ballard A., Holmes H., Young A.R., McMahon S.B. (2009). Ultraviolet-B induced inflammation of human skin: characterisation and comparison with traditional models of hyperlagesia. Eur J Pain.

[8] Blais C., Couture R., Drapeau G., Colman R.W., Adam A. (1997). Involvement of endogenous kinins in the pathogenesis of peptidoglycan-induced arthritis in the Lewis rat. Arthritis Rheum.

[9] Brown H., Moppett I.K., Mahajan R.P. (2003). Transient hyperaemic response to assess vascular reactivity of skin: effect of locally iontophoresed acetylcholine, bradykinin, epinephrine and phenylephrine. Br J Anaesth.

[10] Cervero F., Gilbert R., Hammond R.G.E., Tanner J. (1993). Development of secondary hyperalgesia following non-painful thermal stimulation of the skin: a psychophysical study in man. PAIN®.

[11] Cesare P., Dekker L.V., Sardini A., Parker P.J., McNaughton P.A. (1999). Specific involvement of PKC-ε in sensitization of the neuronal response to painful heat. Neuron.

[12] Cohan V.L., MacGlashan D.W., Warner J.A., Lichtenstein L.M., Proud D. (1991). Mechanisms of mediator release from human skin mast cells upon stimulation by the bradykinin analog, [DArg0-Hyp3-DPhe7]bradykinin. Biochem Pharmacol.

[13] Couture R., Lindsey C.J., Bjorklund A., Quirion R., Hökfelt T. (2000). Brain kallikrein-kinin system: from receptors to neuronal pathways and physiological functions.

[14] Crossman D.C., Fuller R.W. (1988). Bradykinin induced wheal and flare is not mediated by histamine release or cyclooxygenase products. Br J Clin Pharmacol.

[15] Davis C.L., Naeem S., Phagoo S.B., Campbell E.A., Urban L., Burgess G.M. (1996). B-1 bradykinin receptors and sensory neurones. Br J Pharmacol.

[16] Dawes J.M., Calvo M., Perkins J.R., Paterson K.J., Kiesewetter H., Hobbs C., Kaan T.K.Y., Orengo C., Bennett D.L.H., McMahon S.B. (2011). CXCL5 mediates UVB irradiation-induced pain. Sci Transl Med.

[17] de Oliveira Fusaro M.C.G., Pelegrini-da-Silva A., Araldi D., Parada C.A., Tambeli C.H. (2010). P2X3 and P2X2/3 receptors mediate mechanical hyperalgesia induced by bradykinin, but not by pro-inflammatory cytokines, PGE2 or dopamine. Eur J Pharmacol.

[18] Dlamini Z., Bhoola K.D. (2005). Upregulation of tissue kallikrein, kinin B1 receptor, and kinin B2 receptor in mast and giant cells infiltrating oesophageal squamous cell carcinoma. J Clin Pathol.

[19] Dray A., Perkins M. (1993). Bradykinin and inflammatory pain. Trends Neurosci.

[20] Drummond P.D. (2003). Attenuation of axon reflexes to compound 48/80 after repeated iontophoresis of compound 48/80 in skin of the human forearm. Skin Pharmacol Appl Skin Physiol.

[21] Drummond P.D. (2004). The effect of cutaneous mast cell degranulation on sensitivity to heat. Inflamm Res.

[22] Eisenbarth H., Rukwied R., Petersen M., Schmelz M. (2004). Sensitization to bradykinin B1 and B2 receptor activation in UV-B irradiated human skin. PAIN®.

[23] Fadel R., Ramboer I., Chatterjee N., Rihoux J.P., Derde M.P. (2000). Short communication. Cetirizine inhibits bradykinin-induced cutaneous wheal and flare in atopic and healthy subjects. Allergy.

[24] Fathy D.B., Leeb T., Mathis S.A., Leeb-Lundberg L.M.F. (1999). Spontaneous human B2 bradykinin receptor activity determines the action of partial agonists as agonists or inverse agonists. J Biol Chem.

[25] Fruhstorfer H., Lindblom U., Schmidt W.C. (1976). Method for quantitative estimation of thermal thresholds in patients. J Neurol Neurosurg Psychiatry.

[26] Greaves M., Shuster S. (1967). Responses of skin blood vessels to bradykinin, histamine and 5-hydroxytryptamine. J Physiol.

[27] Hamza M., Wang X.M., Adam A., Brahim J.S., Rowan J.S., Carmona G.N., Dionne R.A. (2010). Kinin B1 receptors contributes to acute pain following minor surgery in humans. Mol Pain.

[28] Hawgood B.J. (1997). Maurício Rocha e Silva MD: snake venom, bradykinin and the rise of autopharmacology. Toxicon.

[29] Honing M.L., Smits P., Morrison P.J., Rabelink T.J. (2000). Bradykinin-induced vasodilation of human forearm resistance vessels is primarily mediated by endothelium-dependent hyperpolarization. Hypertension.

[30] Hosogi M., Schmelz M., Miyachi Y., Ikoma A. (2006). Bradykinin is a potent pruritogen in atopic dermatitis: a switch from pain to itch. PAIN®.

[31] Hwang T.-L., Yeh Y.-A., Chern J.-W., Teng C.-M. (2000). Pharmacological characterization of EK112, a new combined angiotensin II and thromboxane A(2) receptor antagonist. Gen Pharmacol.

[32] Janssens A.S., Heide R., den Hollander J.C., Mulder P.G.M., Tank B., Oranje A.P. (2005). Mast cell distribution in normal adult skin. J Clin Pathol.

[33] Jensen K., Tuxen C., Pedersen-Bjergaard U., Jansen I. (1991). Pain, tenderness, wheal and flare induced by substance-P, bradykinin and 5-hydroxytryptamine in humans. Cephalalgia.

[34] Jensen K., Tuxen C., Pedersen-Bjergaard U., Jansen I., Edvinsson L., Olesen J. (1990). Pain, wheal and flare in human forearm skin induced by bradykinin and 5-hydroxytryptamine. Peptides.

[35] Khasar S.G., Miao F.J.P., Gear R.W., Green P.G., Isenberg W.M., Levine J.D. (2002). Sympathetic-independent bradykinin mechanical hyperalgesia induced by subdiaphragmatic vagotomy in the rat. J Pain.

[36] Kindgen-Milles D. (1995). Effects of prostaglandin E2 on the intensity of bradykinin-evoked pain from skin and veins of humans. Eur J Pharmacol.

[37] Koltzenburg M., Kress M., Reeh P.W. (1992). The nociceptor sensitization by bradykinin does not depend on sympathetic neurons. Neuroscience.

[38] Koppert W., Reeh P.W., Handwerker H.O. (1993). Conditioning of histamine by bradykinin alters responses of rat nociceptor and human itch sensation. Neurosci Lett.

[39] Lawrence C.M., Howel D., Shuster S. (1986). Site variation in anthralin inflammation on forearm skin. Br J Dermatol.

[40] Levine J.D., Taiwo Y.O., Collins S.D., Tam J.K. (1986). Noradrenaline hyperalgesia is mediated through interaction with sympathetic postgahglionic neurone terminals rather than activation of primary afferent nociceptors. Nature.

[41] Liang Y.-F., Haake B., Reeh P.W. (2001). Sustained sensitization and recruitment of rat cutaneous nociceptors by bradykinin and a novel theory of its excitatory action. J Physiol.

[42] Magerl W., Westerman R.A., Mohner B., Handwerker H.O. (1990). Properties of transdermal histamine iontophoresis: differential effects of season, gender, and body region. J Investig Dermatol.

[43] Manning D.C., Raja S.N., Meyer R.A., Campbell J.N. (1991). Pain and hyperalgesia after intradermal injection of bradykinin in humans. Clin Pharm Ther.

[44] Marceau F., Hess J.F., Bachvarov D.R. (1998). The B1 receptors for kinins. Pharmacol Rev.

[45] Menke J.G., Borkowski J.A., Bierilo K.K., MacNeil T., Derrick A.W., Schneck K.A., Ransom R.W., Strader C.D., Linemeyer D.L., Hess J.F. (1994). Expression cloning of a human B1 bradykinin receptor. J Biol Chem.

[46] Mizumura K., Sugiura T., Katanosaka K., Banik R., Kozaki Y. (2009). Excitation and sensitization of nociceptors by bradykinin: what do we know?. Exp Brain Res.

[47] Neugebauer V., Schaible H.G., Schmidt R.F. (1989). Sensitization of articular afferents to mechanical stimuli by bradykinin. Pflügers Arch.

[48] Newton D.J., Khan F., Belch J.J. (2001). Assessment of microvascular endothelial function in human skin. Clin Sci.

[49] Norbury T.A., MacGregor A.J., Urwin J., Spector T.D., McMahon S.B. (2007). Heritability of responses to painful stimuli in women: a classical twin study. Brain.

[50] Perkins M.N., Kelly D. (1993). Induction of bradykinin B1 receptors in vivo in a model of ultra-violet irradiation-induced thermal hyperalgesia in the rat. Br J Pharmacol.

[51] Prado G.N., Taylor L., Zhou X., Ricupero D., Mierke D.F., Polgar P. (2002). Mechanisms regulating the expression, self-maintenance, and signaling-function of the bradykinin B2 and B1 receptors. J Cell Physiol.

[52] Batheja P., Thakur R., Michniak B. (2006). Transdermal iontophoresis. Expert Opin Drug Deliv.

[53] Rolke R., Baron R., Maier C., Tölle T.R., Treede R.D., Beyer A., Binder A., Birbaumer N., Birklein F., Bötefür I.C., Braune S., Flor H., Huge V., Klug R., Landwehrmeyer G.B., Magerl W., Maihöfner C., Rolko C., Schaub C., Scherens A., Sprenger T., Valet M., Wasserka B. (2006). Quantitative sensory testing in the german research network on neuropathic pain (DFNS): standardized protocol and reference values. PAIN®.

[54] Schmelz M., Schmidt R., Weidner C., Hilliges M., Torebjork H.E., Handwerker H.O. (2003). Chemical response pattern of different classes of C-nociceptors to pruritogens and algogens. J Neurophysiol.

[55] Seabrook G.R., Bowery B.J., Heavens R., Brown N., Ford H., Sirinathsinghi D.J.S., Borkowski J.A., Hess J.F., Strader C.D., Hill R.G. (1997). Expression of B-1 and B-2 bradykinin receptor mRNA and their functional roles in sympathetic ganglia and sensory dorsal root ganglia neurones from wild-type and B-2 receptor knockout mice. Neuropharmacology.

[56] Segond von Banchet G., Petersen M., Heppelmann B. (1996). Bradykinin receptors in cultured rat dorsal root ganglion cells: influence of length of time in culture. Neuroscience.

[57] Shin J., Cho H., Hwang S.W., Jung J., Shin C.Y., Lee S.-Y., Kim S.H., Lee M.G., Choi Y.H., Kim J., Haber N.A., Reichling D.B., Khasar S., Levine J.D., Oh U. (2002). Bradykinin-12-lipoxygenase-VR1 signaling pathway for inflammatory hyperalgesia. Proc Natl Acad Sci.

[58] Sugiura T., Tominaga M., Katsuya H., Mizumura K. (2002). Bradykinin lowers the threshold temperature for heat activation of vanilloid receptor 1. J Neurophysiol.

[59] Wallengren J., Håkanson R. (1992). Effects of capsaicin, bradykinin and prostaglandin E2 in the human skin. Br J Dermatol.

[60] Waller R.G. (1931). Persistent haematuria. Proc R Soc Med.

[61] Wang H., Kohno T., Amaya F., Brenner G.J., Ito N., Allchorne A., Ji R.-R., Woolf C.J. (2005). Bradykinin produces pain hypersensitivity by potentiating spinal cord glutamatergic synaptic transmission. J Neurosci.

[62] Wotherspoon G., Winter J. (2000). Bradykinin B1 receptor is constitutively expressed in the rat sensory nervous system. Neurosci Lett.

